# Cerebral microbleed assessment on MRI in a memory clinic: Reliability, prevalence, and implications for anti‐amyloid therapy

**DOI:** 10.1002/dad2.70472

**Published:** 2026-09-30

**Authors:** Ernest Privšek, Anna Marseglia, Igor Košutić, Anna Rosenberg, Tobias Granberg, Milica G. Kramberger, Miia Kivipelto, Eric Westman

**Affiliations:** ^1^ Division of Clinical Geriatrics Center for Alzheimer Research Department of Neurobiology, Care Sciences and Society Karolinska Institutet Huddinge Sweden; ^2^ Department of Radiology Institute of Oncology Ljubljana Ljubljana Slovenia; ^3^ Faculty of Medicine University of Ljubljana Ljubljana Slovenia; ^4^ Neuropsychiatric Epidemiology Unit, Department of Psychiatry and Neurochemistry Institute of Neuroscience and Physiology the Sahlgrenska Academy at the University of Gothenburg Mölndal Sweden; ^5^ Department of Diagnostic and Interventional Radiology Slovenj Gradec General Hospital Slovenj Gradec Slovenia; ^6^ Department of Public Health, Lifestyles and Living Environments Finnish Institute for Health and Welfare Helsinki Finland; ^7^ Department of Neuroradiology Karolinska University Hospital Stockholm Sweden; ^8^ Department of Clinical Neuroscience Karolinska Institutet Stockholm Sweden; ^9^ Department of Neurology University Medical Centre Ljubljana Slovenia; ^10^ Ageing Epidemiology Research Unit School of Public Health Imperial College London London UK; ^11^ Theme Inflammation and Aging Karolinska University Hospital Huddinge Sweden; ^12^ Institute of Public Health and Clinical Nutrition University of Eastern Finland Kuopio Finland

**Keywords:** Alzheimer's disease, anti‐amyloid therapy, cerebral microbleeds, memory clinic, observer agreement

## Abstract

**INTRODUCTION:**

Cerebral microbleeds (CMBs) represent an important magnetic resonance imaging (MRI) marker of cerebral small vessel pathology in memory‐clinic populations, where vascular and neurodegenerative pathologies frequently overlap.

**METHODS:**

This cross‐sectional study included 747 patients from a Swedish memory clinic who underwent 3T MRI with susceptibility‐weighted imaging. Two readers assessed CMBs using standardized criteria. Inter‐ and intra‐observer agreement were evaluated before and after protocol refinement. CMB prevalence and burden were analyzed across diagnostic groups and by cerebrospinal fluid amyloid status.

**RESULTS:**

CMBs were present in 28.6% of patients and frequently observed in subjects with mild cognitive impairment or dementia. Inter‐observer agreement improved from κ = 0.71 to κ = 0.82 after protocol refinement. Among amyloid‐positive patients with cognitive impairment, 11.2% exceeded proposed CMB thresholds for anti‐amyloid therapy eligibility.

**DISCUSSION:**

Standardized CMB assessment improves reliability in memory‐clinic populations. Given its growing impact on treatment eligibility and safety, consistent evaluation is essential for patient management.

## Highlights

- Standardizing and refining the assessment of CMBs on MRI improved inter‐observer reliability for the evaluation of these lesions within memory‐clinic populations.
- CMBs were identified in 28.6% of the patient sample and were more prevalent among individuals with MCI and dementia.
- Among amyloid‐positive patients with cognitive impairment, 11.2% exceeded proposed CMB thresholds relevant for anti‐amyloid therapy eligibility.
- Variation in reporting CMBs can influence both therapy selection and safety discussions concerning new anti‐amyloid medications.
- Reliable and reproducible CMB evaluation is vital in clinical practice to provide guidance for clinical decision‐making.

## 1 BACKGROUND

Cerebral microbleeds (CMBs) are chronic intracerebral hemorrhages that appear as punctate areas of signal loss with blooming artifacts on susceptibility‐sensitive magnetic resonance imaging (MRI).^1^ As reliable markers of cerebral small vessel disease (SVD), they have important diagnostic and prognostic significance in cognitive disorders arising from vascular and neurodegenerative diseases.^2^ In memory‐clinic populations, the number and distribution of CMBs can help establish and differentiate causes of cognitive impairment due to SVD, and they are included in the revised Boston Criteria 2.0 for cerebral amyloid angiopathy (CAA).^3^ Beyond diagnostic classification, CMBs influence therapeutic decisions and clinical trial design. It is recommended that patients with high CMB burden be excluded from anti‐amyloid treatment for Alzheimer's disease (AD) due to increased risk for amyloid‐related imaging abnormalities (ARIA).^4^, ^5^ Anti‐amyloid therapies are increasingly being introduced into clinical practice, assessment of CMB burden plays an important role in treatment selection, highlighting the need for reproducible evaluation. To standardize the assessment, several visual rating scales have been developed.^6^, ^7^ However, manual rating remains challenging, with various levels of intra‐ and inter‐rater agreement, particularly in memory clinics where AD and SVD pathology co‐exist. The variability in reported CMB prevalence across memory‐clinic studies^8^, ^9^, ^10^, ^11^, ^12^ reflects both biological and methodological factors. It takes an experienced rater's judgment to distinguish real CMBs from mimics like calcifications, vascular flow voids, or imaging artifacts. Detection sensitivity may be affected by variability in MRI protocols, image quality, and rating scales, while heterogeneity of these cohorts introduces additional interpretive challenges.

The aim of this study was to assess intra‐ and inter‐observer reliability of CMB rating, identify reasons for disagreement between readers, and explore methodological improvements to the workflow in a memory‐clinic population. A secondary objective was to estimate the prevalence and distribution of CMBs in a “real‐world” memory‐clinic cohort, as memory clinics are expected to play a central role in the implementation of anti‑amyloid therapies, and additional reproducible data on CMB prevalence are needed to better inform the integration of these treatments into clinical practice.

**RESEARCH IN CONTEXT**

1. **Systematic review**: CMBs are key biomarkers of SVD and are often present in memory‐clinic populations. Previous studies have demonstrated varying prevalences of cerebral microbleeds and interobserver reliability, depending on magnetic resonance imaging sequences, rating approaches and cohort populations. Emerging anti‐amyloid treatments targeting Alzheimer's dementia (AD) demand precise assessment of CMB burden, as this metric can determine eligibility for such therapies.
2. **Interpretation**: In this large real‐world memory‐clinic cohort, standardization and refinement of CMB assessment increased the agreement and provided better CMB prevalence and burden estimates. Among amyloid‐positive patients with cognitive impairment, a clinically relevant proportion exceeded proposed thresholds that may influence anti‐amyloid therapy eligibility.
3. **Future directions**: A standardized approach to grading CMBs will be important to promote consistency as anti‐amyloid agents are implemented into the routine care of patients at memory clinics.

## 2 METHODS

### 2.1 Participants

This cross‐sectional study included 836 consecutive individuals referred to the memory clinic at Karolinska University Hospital (Stockholm, Sweden) between April 2018 and June 2024. The study is based on a clinical cohort previously reported in biomarker‐focused publications.^13^, ^14^ All patients underwent cognitive assessments, standardized brain MRI, and cerebrospinal fluid (CSF) biomarkers of AD pathology and clinical data. Exclusion criteria were incomplete or technically inadequate susceptibility‐weighted imaging (SWI) precluding CMB interpretation (*n* = 46) and missing key clinical and demographic information (*n* = 2). Patients were excluded if cognitive symptoms were primarily attributed to a significant psychiatric illness or alcohol abuse (*n* = 11) or if relevant coexisting neuropsychiatric comorbidities were present (*n* = 30). After exclusion, 747 individuals remained (Figure 1).

**FIGURE 1:**
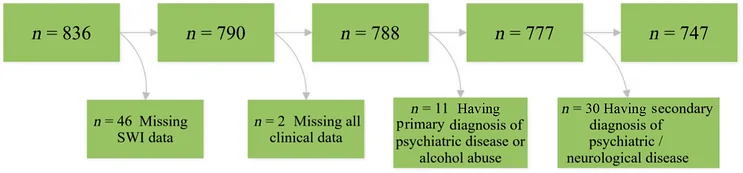
Study flow diagram showing patient inclusion and exclusion.

### 2.2 Diagnostic groups, cognitive assessment, and comorbid disorders

Diagnoses were established clinically according to the International Classification of Diseases‐10th revision (ICD 10) criteria. We identified three groups: subjective cognitive complaints (SCC), mild cognitive impairment (MCI), and dementia. Briefly, SCC included patients with reported cognitive concerns, but without dementia or objective cognitive impairment (ICD‐10 codes R41.8, R41.3). MCI included individuals without dementia but with objective cognitive impairment (ICD‐10 code F06.7). The dementia group included all major neurocognitive disorders, combining AD, vascular, and other types (ICD‐10 codes F00–F03, F01.2–F01.3, F01.9, F02.0–F02.1, F02.3, F02.8, F03.9, and G30.0). Cognition was assessed by a multidisciplinary team and included Mini‐Mental State Examination (MMSE) and Montreal Cognitive Assessment (MoCA) total scores. Comorbidities were determined from medical records, with stroke defined as a radiologically confirmed and documented event. Hypertension was defined as systolic blood pressure ≥ 140 mmHg and/or a diastolic blood pressure ≥ 90 mmHg, based on measurements obtained during the visit. HbA1c values were used as a proxy for diabetes mellitus, defined as HbA1c ≥ 6.5% (≥48 mmol/mol) according to American Diabetes Association criteria.^15^ Apolipoprotein E genotype was dichotomized as ε4 carrier (at least one ε4 allele) versus non‐carriers. Amyloid positivity was determined using the CSF Aβ42/Aβ40 × 10 ratio, with platform‐specific cutoffs of <0.60 for the Innotest assays and <0.86 for the Lumipulse G‐series platform (Fujirebio Europe, Ghent, Belgium).^16^

### 2.3 MRI acquisition

All participants underwent brain MRI on the same clinical whole‐body 3 Tesla (T) scanner (Discovery MR750 updated during the study to a Signa Premier; GE Medical Systems, Milwaukee, WI, USA) according to the Department of Neuroradiology's standardized neurodegenerative protocol, which included sagittal three‐dimensional (3D) T1‐weighted fluid‐attenuated inversion recovery (FLAIR), axial 3D T1‐weighted brain volume imaging (BRAVO), sagittal 3D T2‐weighted FLAIR CUBE, axial 2D T2‐weighted motion‐corrected periodically rotated overlapping parallel lines with enhanced reconstruction (PROPELLER), axial 2D diffusion‐weighted imaging, and axial 3D SWI sequences. For the SWI sequence specifically, acquisition parameters varied across protocol versions used during the study period (echo time range, 20 to 28 ms; repetition time range, 30 to 45 ms; slice thickness, 2 mm). Thin‐slab minimum‐intensity projections (mIP) were reconstructed for interpretation.

### 2.4 Rating protocol and image analysis

MRI ratings were completed before application of the final patient‐level exclusion criteria and were independently reviewed by two readers, a board‐certified radiologist (reader 1. I.K.) and a trained radiology resident (reader 2, E.P.), both blinded to clinical information. Prior to formal rating, the readers jointly reviewed and agreed upon a standardized rating protocol. The presence and number of CMBs were assessed on SWI according to current consensus criteria outlined in the STandards for ReportIng Vascular changes on nEuroimaging (STRIVE)^1^ with microbleed classification and exclusion of mimics performed according to Microbleed Anatomical Rating Scale (MARS)^7^ and anatomical categorization based on a simplified version of MARS.^17^ A set of 200 randomly selected MRI scans was rated independently to establish baseline consistency and determine sources of disagreement. Discrepancies and ambiguous findings were reviewed jointly, resulting in refinement and clarification of working definitions for CMB identification. The finalized protocol (Table 1) was then applied independently to the entire dataset. CMBs were defined as small (≤10 mm), homogeneous, markedly hypointense (“black”), round or ovoid foci on SWI, clearly distinct from calcification, vascular flow voids, or other artifacts. Lesions not meeting core criteria were regarded as indeterminate and were excluded if they lacked morphology suggestive of a focal hemorrhage, were not visible on at least two adjacent slices, or showed continuity with the venous structures. Lesions smaller than nearby cortical veins (approximately 1 mm) were discarded as likely subresolution artifacts. Small “gray” lesions located adjacent to the ventricles, basal ganglia, skull base, or dural sinuses were excluded because of frequent susceptibility artifacts and possible partial volume effects in these regions. In the basal ganglia, where calcifications and depositions of paramagnetic substances are common, lesions were accepted if they met core criteria for CMB and showed a clear asymmetry between hemispheres. Phase maps were used systematically to distinguish calcifications from true CMBs, and lesions not clearly visible on phase images were generally excluded. Lesions fulfilling the core criteria were additionally excluded if they were spatially associated with large infarcts, macrohemorrhages, or focal post‐traumatic lesions, where susceptibility effects likely represented secondary hemorrhagic changes rather than sporadic CMBs. Thick mIP reconstructions of SWI data were generated to enhance the conspicuity of small CMBs and improve inter‐rater consistency. We used RadiAnt DICOM Viewer (version 2020.2) to evaluate the MRI scans.

**TABLE 1:** Final operational criteria and decision rules for CMB ratings on SWI.

Domain	Criterion	Operational definition / decision rule
**Conceptual definition**	Consensus definitions	STRIVE‐based definitions of CMBs
**Anatomical classification**	Location	Categorized using a simplified Microbleed Anatomical Rating Scale (MARS) scheme
**Operational definition** **(MARS)**	Size	≤ 10 mm in maximum diameter
	Signal	Homogeneous, markedly hypointense (“black”) focus on SWI; not seen well on other sequences
	Shape	Round or ovoid
**Minimum size threshold**	Subresolution exclusion	Lesions smaller than adjacent cortical veins (∼1 mm) on the same slice were excluded
**Indeterminate lesion evaluation**	Definition	Lesions not fulfilling all core CMB criteria (e.g., borderline size, shape, heterogeneous, or mildly hypointense “gray” signal)
	Morphology exclusion	Indeterminate lesions with irregular, non–round/ovoid morphology or poorly defined margins were excluded.
	Slice‐consistency exclusion	Indeterminate lesions not visible on at least two adjacent slices
	Venous continuity exclusion	Indeterminate lesions continuous with cortical or medullary veins
	Location‐based exclusion	Indeterminate lesions adjacent to ventricles, basal ganglia, skull base, or dural sinuses were excluded because of frequent susceptibility artifacts in these regions
**Etiology‐based exclusion**	Secondary hemorrhagic context	Lesions fulfilling all core CMB criteria were excluded if spatially associated with large infarcts, macro‐hemorrhages, or focal post‐traumatic lesions, where susceptibility effects likely represent secondary hemorrhage rather than sporadic CMBs
**Basal ganglia**	Regional specificity	Lesions accepted only if >1 mm and demonstrating clear hemispheric asymmetry
**Phase imaging**	Susceptibility source confirmation	Phase images used routinely; indeterminate lesions without supportive phase information were generally excluded
**Image reconstruction**	mIP use	Thick minimum‐intensity projections generated to enhance CMB conspicuity

After independent ratings the reader with fewer identified CMBs re‐examined the discrepant scans, and remaining disagreements were resolved by joint consensus review. Interrater reliability was assessed twice: an initial agreement based on the first 200 scans before protocol refinement, and final inter‐rater agreement, based on the complete dataset rated with the final criteria. Intra‐rater reliability was tested by comparing each reader's initial and final ratings of the same 200 scans and a separate retest, which was derived from 30 randomly selected cases that were re‐rated at the study's end. The complete workflow is summarized in Figure 2.

**FIGURE 2:**
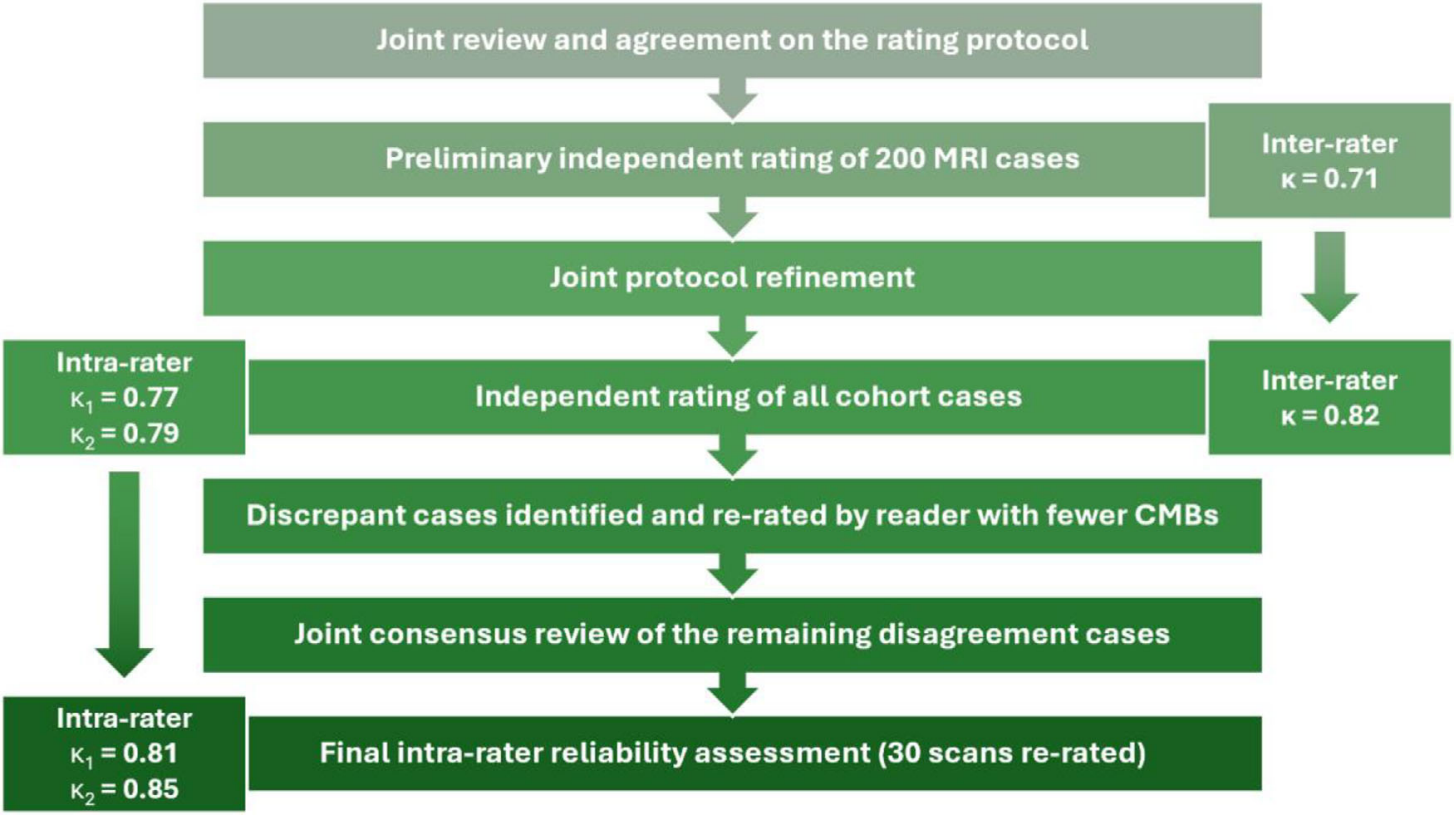
Workflow for protocol agreement and refinement, cerebral microbleed rating, and consensus procedure with inter‐ and intra‐rater agreements.

### 2.5 Statistical analysis

Intra‐ and inter‐observer agreement for CMB burden categories (0, 1, 2 to 4, 5 to 9, ≥10) was evaluated with Cohen's κ with quadratic weighting and 95% confidence level, κ values of 0.00 to 0.20 indicated slight agreement, 0.21 to 0.40 fair agreement, 0.41 to 0.60 moderate agreement, 0.61 to 0.80 substantial agreement, and 0.81 to 1.00 excellent agreement. The change in quadratic‐weighted inter‐rater κ after protocol refinement was assessed in scans with complete ratings before and after rating protocol refinement using paired subject‐level bootstrapping with 5000 resamples; the κ difference and normal‐based 95% bootstrap confidence interval were estimated. Continuous variables are reported as medians with interquartile ranges and categorical variables as frequencies with percentages. Data are given as median with interquartile range for continuous variables and frequency with percentage for categorical variables. Normality was tested for all continuous variables; because distributions were non‐normal, group comparisons used the Kruskal–Wallis test. Pearson's χ^2^ was applied to categorical variables, including CMB prevalence comparisons across diagnostic groups. Fisher's exact test was used when cell counts were sparse. Adjusted analyses used linear regression for continuous outcomes, modified Poisson regression for binary outcomes, and multinomial or ordinal logistic regression for CMB burden categories, all with robust standard errors, as appropriate. Covariates are specified in the corresponding table footnotes. A two‑sided *p* < 0.05 was considered statistically significant. Statistical analyses were performed in Stata‐SE (version 18; StataCorp).

## 3 RESULTS

### 3.1 Study population and dataset characteristics

The study included 747 individuals; 421 (56.4%) were women, 390 (52.2%) had SCC, 195 (26.1%) had MCI, and 162 (21.7%) had dementia (Table 2). The median age was 60 years (IQR: 56 to 63) and differed significantly across diagnostic groups (*p* < 0.001). After adjustment, APOE ε4 carrier frequency also differed across groups (*p* = 0.006), increasing from 40.9% in SCC to 48.2% in MCI and 56.9% in dementia. Education differed modestly across groups (*p* = 0.026), whereas cognitive performance showed a clear gradient, with progressively lower MoCA and MMSE scores from SCC to MCI and dementia (both *p* < 0.001). Stroke prevalence, HbA1c levels, and the prevalence of HbA1c ≥6.5% also differed across diagnostic groups (*p* = 0.002, *p* = 0.006, and *p* = 0.007, respectively), whereas sex distribution and hypertension did not remain significantly different after adjustment (*p* = 0.067 and *p* = 0.099).

**TABLE 2:** Baseline demographic, cognitive, vascular, and MRI characteristics by diagnostic Group.

Characteristics	All (*n* = 747)	SCC (*n* = 390)	MCI (*n* = 195)	Dementia (*n* = 162)	*p* value	Adjusted *p* value
**Age, years**	60 (56 to 63)	58 (54 to 62)	62 (57 to 64)	62 (59 to 65)	<0.001	<0.001
**Female, n (%)**	421 (56.4)	243 (62.3)	91 (46.7)	87 (53.7)	0.001	0.067
**APOE ε4 carrier, *n* (%)**	304 (46.3)	140 (40.9)	82 (48.2)	82 (56.9)	0.005	0.006
**Pathological Aβ42/40 ratio, *n* (%)**	216 (38.8)	64 (21.8)	66 (46.5)	86 (71.7)	<0.001	<0.001
**Cognition**						
Education, years	13 (11 to 16)	14 (12 to 16)	12 (11 to 16)	13 (11 to 15)	0.014	0.026
MoCA score	24 (20 to 26)	26 (24 to 28)	23 (21 to 25)	17 (14 to 20)	<0.001	<0.001
MMSE score	27 (24 to 29)	28 (26 to 29)	27 (24 to 29)	22 (19 to 26)	<0.001	<0.001
**CMBs, *n* (%)**						
Present (at least one)	214 (28.6)	93 (23.8)	67 (34.4)	54 (33.3)	0.010	0.799
1	121 (16.2)	61 (15.6)	33 (16.9)	27 (16.7)	0.008	0.187
2 to 4	53 (7.1)	26 (6.7)	16 (8.2)	11 (6.8)		
5 to 9	12 (1.6)	3 (0.8)	5 (2.6)	4 (2.5)		
≥10	28 (3.7)	3 (0.8)	13 (6.7)	12 (7.4)		
Deep only	19 (2.5)	5 (1.3)	10 (5.1)	4 (2.5)	0.023	.
**Comorbidities**						
Hypertension, *n* (%)	357 (51.0)	165 (45.2)	107 (59.1)	85 (55.2)	0.005	0.099
Stroke, *n* (%)	106 (14.6)	31 (8.1)	44 (23.0)	31 (20.4)	<0.001	0.002
HbA1c, %	5.54 (5.26 to 5.72)	5.45 (5.26 to 5.72)	5.54 (5.35 to 5.81)	5.54 (5.35 to 5.90)	0.002	0.006
HbA1c ≥6.5%, *n* (%)	39 (5.8)	9 (2.5)	15 (8.6)	15 (10.9)	<0.001	0.007

*Note*: Data are presented as median (25th to 75th percentile) or *n* (%). Unadjusted *p* values were calculated using Kruskal–Wallis tests for continuous variables, Pearson χ^2^ tests for categorical variables, and the Fisher–Freeman–Halton exact test for deep‐only CMBs. Adjusted *p* values compare SCC, MCI, and dementia after accounting for the following covariates: age: sex; female sex: age and APOE ε4; APOE4 carrier status: age and sex; pathological Aβ42/40 ratio: age, sex, and APOE4; education: age and sex; MoCA and MMSE: age, sex, education, and APOE4; CMB presence and burden: age, sex, hypertension, and APOE4; hypertension and HbA1c outcomes: age and sex; and stroke: age, sex, and hypertension. Deep‐only CMBs were not adjusted because of the small number of events.

CSF Aβ42/Aβ40 status was available for 556 of 747 participants (SCC, *n* = 294; MCI, *n* = 142; dementia, *n* = 120); percentages for pathological Aβ42/Aβ40 ratio were calculated using these available‐case denominators. Other missing data: APOE ε4, 91; education, 147; MoCA, 143; MMSE, 296; stroke, 21; hypertension, 47; HbA1c, 73. Two‐sided *p* < 0.05 indicated statistical significance.

Abbreviations: APOE ε4, apolipoprotein E ε4 allele; Aβ, amyloid beta; CMB, cerebral microbleed; HbA1c, glycated hemoglobin; MCI, mild cognitive impairment; MMSE, Mini‐Mental State Examination; MoCA, Montreal Cognitive Assessment; SCC, subjective cognitive complaints.

After adjustment, diagnostic‐group distribution did not differ significantly by sex (*p* = 0.056; Table 3). Men had higher prevalences of hypertension (57.3% vs 46.1%; *p* = 0.007), stroke (21.4% vs 9.3%; *p* < 0.001), and HbA1c‐defined diabetes (8.2% vs 3.9%; *p* = 0.036). APOE ε4 carrier status and cognitive performance did not differ by sex.

**TABLE 3:** Characteristics by sex at birth.

Characteristics	All (*n* = 747)	Female (*n* = 421)	Male (*n* = 326)	*p* value	Adjusted *p* value
**Age, years**	60 (56 to 63)	60 (56 to 63)	61 (57 to 64)	0.012	.
**APOE ε4 carrier, *n* (%)**	304 (46.3)	167 (45.0)	137 (48.1)	0.436	0.493
**Pathological Aβ42/40 ratio, *n* (%)**	216 (38.8)	125 (39.3)	91 (38.2)	0.797	0.332
**Diagnostic group, *n* (%)**					
Subjective cognitive complaints	390 (52.2)	243 (57.7)	147 (45.1)	0.001	0.056
Mild cognitive impairment	195 (26.1)	91 (21.6)	104 (31.9)		
Dementia	162 (21.7)	87 (20.7)	75 (23.0)		
**Cognition**					
Education, years	13 (11 to 16)	13 (11 to 16)	13 (11 to 16)	0.399	0.952
MoCA score	24 (20 to 26)	24 (20 to 27)	24 (20 to 26)	0.470	0.614
MMSE score	27 (24 to 29)	27 (24 to 29)	27 (23 to 29)	0.884	0.929
**CMBs, *n* (%)**					
Present (at least one)	214 (28.6)	91 (21.6)	123 (37.7)	<0.001	0.001
1	121 (16.2)	55 (13.1)	66 (20.2)	0.557	0.470
2 to 4	53 (7.1)	22 (5.2)	31 (9.5)		
5 to 9	12 (1.6)	3 (0.7)	9 (2.8)		
≥10	28 (3.7)	11 (2.6)	17 (5.2)		
Deep only	19 (2.5)	7 (1.7)	12 (3.7)	0.102	.
**Comorbidities**					
Hypertension, *n* (%)	357 (51.0)	181 (46.1)	176 (57.3)	0.003	0.007
Stroke, *n* (%)	106 (14.6)	38 (9.3)	68 (21.4)	<0.001	<0.001
HbA1c, %	5.54 (5.26 to 5.72)	5.45 (5.26 to 5.72)	5.54 (5.35 to 5.81)	0.024	0.375
HbA1c ≥6.5%, *n* (%)	39 (5.8)	15 (3.9)	24 (8.2)	0.018	0.036

*Note*: Data are presented as median (25th to 75th percentile) or *n* (%). Unadjusted *p* values were calculated using Kruskal–Wallis tests for continuous variables, Pearson χ^2^ tests for categorical variables, and Fisher exact tests for deep‐only CMBs. Adjusted *p* values compare women and men after accounting for the following covariates: APOE ε4 carrier status: age; pathological Aβ42/40 ratio: age and APOE ε4; diagnostic group: age and APOE4; education: age; MoCA and MMSE: age, education, and APOE4; CMB presence and burden: age, hypertension, and APOE4; hypertension and HbA1c outcomes: age; and stroke: age and hypertension. Age was not adjusted. Deep‐only CMBs were not adjusted because of the small number of events. Two‐sided *p* < 0.05 indicated statistical significance.

CSF Aβ42/Aβ40 status was available for 556 of 747 participants (women, *n* = 318; men, *n* = 238); percentages for pathological Aβ42/Aβ40 ratio were calculated using these available‐case denominators. Other missing data: APOE ε4, 91; education, 147; MoCA, 143; MMSE, 296; stroke, 21; hypertension, 47; HbA1c, 73.

Abbreviations: APOE ε4, apolipoprotein E ε4 allele; Aβ, amyloid beta; CMB, cerebral microbleed; HbA1c, glycated hemoglobin; MCI, mild cognitive impairment; MMSE, Mini‐Mental State Examination; MoCA, Montreal Cognitive Assessment; SCC, subjective cognitive complaints.

CSF Aβ42/Aβ40 status was available for 556 participants and 216 (38.8%) had a pathological ratio (Table 4). Amyloid positivity differed across diagnostic groups after adjustment (*p* < 0.001), with prevalences of 21.8% in SCC, 46.5% in MCI, and 71.7% in dementia. Amyloid‐positive participants were more often APOE ε4 carriers (69.0% vs 33.6%; *p* < 0.001) and had lower MoCA and MMSE scores (both *p* < 0.001).

**TABLE 4:** Characteristics of study subsample based on CSF amyloid β42/40 ratios.

Characteristics	All (*n* = 556)	Pathological Aβ42/40 ratio *n* = 216	Normal Aβ42/40 ratio *n* = 340	*p* value	Adjusted *p* value
**Age, years**	60 (56 to 63)	61 (58 to 64)	59 (55 to 62)	<0.001	.
**Female, *n* (%)**	318 (57.2)	125 (57.9)	193 (56.8)	0.797	.
**APOE ε4 carrier, *n* (%)**	256 (47.4)	145 (69.0)	111 (33.6)	<0.001	<0.001
**Diagnostic group, *n* (%)**					
Subjective cognitive complaints	294 (52.9)	64 (29.6)	230 (67.6)	<0.001	<0.001
Mild cognitive impairment	142 (25.5)	66 (30.6)	76 (22.4)		
Dementia	120 (21.6)	86 (39.8)	34 (10.0)		
**Cognition**					
Education, years	13 (12 to 16)	13 (12 to 16)	13 (11 to 16)	0.795	0.748
MoCA score	24 (19 to 27)	22 (17 to 25)	25 (22 to 27)	<0.001	<0.001
MMSE score	27 (24 to 29)	26 (22 to 29)	28 (25 to 29)	<0.001	<0.001
**CMBs, *n* (%)**					
Present (at least one)	152 (27.3)	60 (27.8)	92 (27.1)	0.853	0.402
1	87 (15.6)	26 (12.0)	61 (17.9)	0.023	0.117
2 to 4	34 (6.1)	16 (7.4)	18 (5.3)		
5 to 9	10 (1.8)	7 (3.2)	3 (0.9)		
≥10	21 (3.8)	11 (5.1)	10 (2.9)		
Deep only	13 (2.3)	3 (1.4)	10 (2.9)	0.388	.
**Comorbidities**					
Hypertension, *n* (%)	272 (51.1)	119 (56.7)	153 (47.5)	0.039	0.148
Stroke, *n* (%)	82 (14.9)	37 (17.3)	45 (13.4)	0.216	0.712
HbA1c, %	5.45 (5.26 to 5.72)	5.45 (5.26 to 5.72)	5.45 (5.26 to 5.72)	0.813	0.503
HbA1c ≥6.5%, *n* (%)	30 (5.8)	13 (6.3)	17 (5.4)	0.652	0.939

*Note*: Data are presented as median (25th to 75th percentile) or *n* (%). Unadjusted *p* values were calculated using Kruskal–Wallis tests for continuous variables, Pearson χ^2^ tests for categorical variables, and Fisher exact tests for deep‐only CMBs. Adjusted *p* values compare amyloid‐positive and amyloid‐negative participants after accounting for the following covariates: APOE ε4 carrier status: age; MCI versus dementia diagnosis: age and sex; education: age and sex; MoCA and MMSE: age, sex, and education; CMB presence and burden: age, sex, and hypertension; hypertension and HbA1c outcomes: age and sex; and stroke: age, sex, and hypertension. Age and female sex were not adjusted. Deep‐only CMBs were not adjusted because of the small number of events. The CMB‐burden *p* value shown is the global comparison across 1, 2 to 4, 5 to 9, and ≥10 CMBs among CMB‐positive participants. Two‐sided *p* < 0.05 indicated statistical significance.

Missing data: APOE ε4, 16; education, 103; MoCA, 95; MMSE, 203; stroke, 7; hypertension, 24; HbA1c, 36.

Abbreviations: Aβ, amyloid beta; APOE ε4, apolipoprotein E ε4 allele; CMB, cerebral microbleed; HbA1c, glycated hemoglobin; MCI, mild cognitive impairment; MMSE, Mini‐Mental State Examination; MoCA, Montreal Cognitive Assessment; SCC, subjective cognitive complaints.

A clinically relevant subgroup analysis (Table S1) included 262 participants with MCI or dementia and available CSF amyloid status, 152 (58.0%) were amyloid‐positive and 110 (42.0%) amyloid‐negative. Amyloid‐positive participants were more frequently APOE ε4 carriers (68.0% vs 30.8%; adjusted *p* < 0.001) and more often had dementia (56.6% vs 30.9%; adjusted *p* < 0.001).

### 3.2 CMBs detection

CMBs were detected in 214 of 747 participants (28.6%, presented in Table 2). CMB prevalence was 23.8% in SCC, 34.4% in MCI, and 33.3% in dementia, but after adjustment neither CMB prevalence nor burden differed across diagnostic groups (*p* = 0.799 and *p* = 0.187, respectively). CMB prevalence was higher in men than women (37.7% vs 21.6%; adjusted *p* = 0.001), whereas CMB burden among CMB‐positive participants did not differ by sex (adjusted *p* = 0.470).

In the whole‐cohort amyloid‐stratified analysis, the prevalence of CMBs was similar (27.8% vs 27.1%; adjusted *p* = 0.402), and the distribution of CMB burden categories did not differ (adjusted *p* = 0.117). However, unadjusted analysis (*p* = 0.023) showed a shift toward higher burden in amyloid‐positive participants. Among patients with a clinical diagnosis of MCI or dementia CMB prevalence was similar in amyloid‐positive and amyloid‐negative participants (30.9% vs 32.7%; adjusted *p* = 0.598), as was CMB burden (adjusted *p* = 0.747). Among the 152 amyloid‐positive participants with MCI or dementia, 17 (11.2%) had ≥4 CMBs.

### 3.3 Inter‐ and intra‐observer agreement

In a preliminary test sample (*n* = 200), inter‐observer agreement for CMB presence and count was good (κ = 0.71). Following joint review of borderline cases to harmonize the application of STRIVE and MARS criteria, the entire cohort was re‐rated and the resulting inter‐observer reliability was excellent (κ = 0.82). Of the 200 scans selected for preliminary assessment, 187 had complete paired ratings before and after protocol refinement. To determine whether this improvement in inter‐observer kappa was statistically significant, we performed a paired bootstrap comparison in the 187 scans. In this paired subset, κ increased from 0.714 to 0.841 (Δκ = 0.127; 95% CI: 0.020 to 0.235; *p* = 0.020). Intra‐observer consistency for the 200 MRI scans re‐rated following protocol refinement was good (κ = 0.77 and 0.79 for readers 1 and 2) and excellent (κ = 0.81 and 0.85) for a random subsample of 30 cases re‐rated at study completion. Inter‐ and intra‐rater agreement and the study workflow are summarized in Figure 2.

## 4 DISCUSSION

Although there are established consensus frameworks to standardize CMB definitions and structured approaches to their identification and anatomical categorization, each MRI dataset has its own characteristics due to variability in MRI sequence parameters, scanner performance, and mix of underlying pathologies. Therefore, supplementing compliance with published standards, joint evaluation of cohort‐specific properties, and cyclical patterns of improvement of rating rules among multiple readers can improve the reliability and reproducibility of CMB assessment. The full multistep workflow may be too resource‐intensive for routine reporting, yet several elements are transferable to clinical practice. Radiology departments could establish local CMB definitions aligned with STRIVE and MARS and periodically calibrate them through independent review of a representative subsample followed by consensus discussion, particularly after changes in scanner hardware, acquisition, or reconstruction. Where available, source images should be reviewed alongside thick‐slab mIP reconstructions and phase images to improve detection and exclude calcification or other non‐hemorrhagic susceptibility effects. Rather than dual reading every scan, second‐reader or consensus review could be reserved for borderline lesions, uncertain morphology, high‐burden examinations, or counts near clinically relevant thresholds.

Reviewing the preliminary MRI ratings, most disagreements concerned small inhomogeneous or low‐conspicuity lesions, near the cortical surface, periventricular regions, or skull base. The difference in counts between the two readers increased in higher CMB cases, suggesting heteroscedasticity in error measurement. This pattern is likely due to the increasing perceptual and cognitive difficulty of counting many small lesions, missing or double counting of single foci. High‐burden instances are prone to error in distinguishing between tightly adjacent or confluent microbleeds (Figure S1). These findings underscore the utility of counting aids or computer‐assisted detection pipelines to enable consistent and reliable lesion counts.^18^ A further source of disagreement was highlighted in those with low CMB burden (one or two small lesions), where one reader could miss all microbleeds. Borderline instances such as these can have a marked bias on prevalence estimations and emphasize the need for double independent readings to prevent false negatives. Where a single‐reader approach is unavoidable, the use of computer‐aided or AI‐based detection software may minimize oversight.

Today, CMB detection software has progressed from semi‐automated candidate‐generation tools requiring manual correction to deep‐learning models optimized for lesion detection and reducing false positives.^19^, ^20^ AI algorithms for chronic CMB detection achieved pooled sensitivity, specificity, and accuracy of 91.6%, 93.9%, and 92.7%, respectively.^21^ Further, AI assistance increased per‐lesion sensitivity for all readers.^22^ However, high sensitivity alone is insufficient for routine use, which has driven recent approaches incorporating false‐positive reduction, anatomical localization, multimodal inputs, and post‐processing strategies.^19^, ^20^, ^23^, ^24^ Moreover, real‐world implementation remains limited by reduced performance after external validation, imperfect generalization across gradient echo sequences (GRE) and SWI, integration into picture archiving and communication system (PACS)/reporting workflows, deployment complexity, and the need for local validation and post‐deployment monitoring.^25^, ^26^

Across both the preliminary and final intra‑rater assessments, agreement improved for both readers, reflecting excellent consistency after protocol optimization and increased familiarity with the dataset. Our results are in line with values reported in similar cohorts. Ikeda et al.^27^ reported a weighted kappa of 0.83 between an experienced neuroradiologist and radiologist. Similar results were observed with a kappa of 0.75 in an Australian memory‐clinic cohort.^8^ It should be noted that a single reported reliability metric is not consistently capable of capturing the full nature of reader agreement, particularly where counts, lesion locations, or ordinal burden categories are considered.^28^ In this study, κ was calculated for ordinal counts of CMB using quadratic weighting, an intermediate approach between binary classification and continuous count data.

Reliable CMB assessment is clinically relevant across several disorders. In population‐based cohorts, CMB presence and increasing CMB burden are associated with higher stroke risk, whereas lobar CMBs, reflecting an amyloid‐angiopathy pattern, identify individuals at increased risk of intracerebral hemorrhage.^29^ In individuals with atrial fibrillation receiving anticoagulation after ischemic stroke or transient ischemic attack, CMB presence and increasing burden are associated with a higher risk of symptomatic intracranial hemorrhage, with ≥5 CMBs identifying a particularly high‐risk subgroup. Nevertheless, CMBs should inform an individualized assessment of ischemic and hemorrhagic risks rather than serve as an isolated reason to withhold anticoagulation.^2^ CMBs also inform hemorrhagic‐risk assessment before intravenous thrombolysis for acute ischemic stroke, particularly when the CMB burden is high.^2^, ^30^ In memory‐clinic practice, strictly lobar CMBs support the diagnosis of CAA and may help characterize vascular contributions to cognitive impairment.^2^, ^3^ These clinical uses all depend on accurate lesion counts and anatomical classification, making methodological differences in MRI acquisition and rating particularly important.

The prevalence of CMBs in memory‐clinic studies^8^, ^9^, ^10^, ^11^, ^12^ varies from approximately 15% to over 40%. This variation can be explained by differences in MRI field strength, type of sequence, rating method, and heterogeneous patient cohorts. Highly cited studies^31^, ^32^ were performed over a decade ago with 1.5T T2* imaging, which underestimates lesion burden compared to state‐of‐the‐art 3T SWI protocols.^9^, ^11^ In our sample, CMBs were found in 28.6% of individuals, a frequency comparable to recent memory‐clinic studies. Wrigley et al.^8^ found CMBs in 27% of individuals in a memory‐clinic cohort with a similar age distribution, 32% in those with MCI, and 39% in dementia. The authors reported higher detection on 3T MRI (31%) than on 1.5T (22%), as expected with increased susceptibility effects at higher field strength.

CMB prevalence and burden differed across diagnostic groups in unadjusted analyses, with higher values in the MCI and dementia groups than in the SCC group. However, these differences were no longer statistically significant after adjustment for age, sex, hypertension, and APOE ε4 status. The unadjusted pattern may therefore reflect demographic and vascular differences across diagnostic groups rather than an independent association between diagnostic status and CMBs. Indeed, we observed a higher prevalence of CMBs in men than in women. This finding is consistent with the general pattern reported in large population‐based and stroke cohorts, in which men more frequently exhibit CMBs, particularly those associated with deep or hypertensive SVD.^33^ However, burden‐category distributions were similar between the sexes, suggesting that male sex was associated primarily with CMB presence rather than greater accumulation among CMB‐positive individuals. Longer cumulative exposure to hypertension and sex‐related differences in cerebral SVD phenotype have been proposed as possible explanations for the male excess. Nevertheless, hypertension in our study was defined using blood pressure measured at the clinical assessment and therefore represents only a proxy that does not capture lifetime exposure, previous diagnosis, treatment, or blood‐pressure control. Residual vascular and clinical confounding consequently cannot be excluded.

Although the overall prevalence of CMBs was similar between amyloid‐positive and amyloid‐negative participants, amyloid‐positive individuals showed a greater unadjusted CMB burden. However, this association was attenuated after adjustment for age, sex, and hypertension and was no longer statistically significant. This suggests that the unadjusted difference may partly reflect demographic and vascular differences between the amyloid groups rather than an independent effect of amyloid positivity.

Our data translate into clinical practice, especially given the newly emerging anti‐amyloid monoclonal antibodies, such as lecanemab^34^ and, more recently, donanemab.^35^ For both treatments, the presence of cerebral microhemorrhages is an important safety‐related exclusion criterion (cut‐off > 4 being proposed in the appropriate use recommendations for lecanemab and donanemab^4^, ^5^). In our patient cohort, 5.4% had more than four microbleeds, representing 18.7% of all CMB‐positive patients. Among all amyloid‐positive patients with a clinical diagnosis of MCI or dementia (potential candidate treatment), 11.2% met this exclusion criterion. Recent studies highlight that eligibility for anti‐amyloid treatment may represent a substantial challenge when implementing these therapies in routine clinical practice, even in memory clinics with comprehensive diagnostic infrastructure and established national guidelines for dementia evaluation.^13^, ^14^ Supporting this, a study^36^ in a relatively young memory‐clinic population comparable to ours reported that nearly one‐third of patients with clinical MCI or AD dementia and confirmed amyloid pathology were excluded from anti‐amyloid therapy due to a high CMB burden (>4), underscoring the significant impact of radiological exclusion criteria on real‐world treatment eligibility.

In this study, we systematically evaluated the validity of CMB ratings and identified key sources of inter‐ and intra‐rater variability. Our findings indicate that independent dual reading, a consensus review, and ongoing refinement of the rating protocol significantly enhanced the reliability of CMB assessment in this cohort. Meanwhile, the burden of CMBs was explored in terms of their prevalence among patient populations, including a subset of potential amyloid‐therapy candidates with significant burdens of the type that could potentially affect therapy eligibility. These observations suggest that the use of a standardized, reliable procedure for identifying CMB may support consistent eligibility criteria while accounting for patient safety.

## CONFLICT OF INTEREST STATEMENT

The authors declare no conflicts of interest. Author disclosures are available in the Supporting Information.

## CONSENT STATEMENT

The Karolinska University Hospital electronic database and biobank for clinical research (GEDOC), and this study received ethical approval (Regional Ethical Review Board in Sweden; registration nos. 2011/1987‐31/4, 2020‐06484, and 2021‐04428). All participants provided written informed consent.

## Supporting information



Supporting Information

Supporting Information

Supporting Information
