# Supplementary material for: Cerebral microbleed assessment on MRI in a memory clinic: Reliability, prevalence, and implications for anti‐amyloid therapy
Source: Alzheimers Dement (Amst). 2026 Sep 30;18(4):e70472. doi: 10.1002/dad2.70472 (PMC13627262; doi:10.1002/dad2.70472)
Supplement: Supplementary file 2 [file DAD2-18-e70472-s001.docx]

Supplementary table 1: Characteristics of the MCI and dementia sub-sample based on CSF amyloid β42/40 ratios

| **Characteristics** | **All (n=262)** | **Pathological**  **Aβ42/40 ratio (n=152)** | **Normal Aβ42/40 ratio (n=110)** | **P value** | **Adjusted P value** |
| --- | --- | --- | --- | --- | --- |
| Age, years | 62 (58-64) | 62 (58-64) | 61 (57-64) | 0.117 | . |
| Female, n (%) | 135 (51.5) | 85 (55.9) | 50 (45.5) | 0.094 | . |
| APOE4 carrier, n (%) | 133 (52.4) | 100 (68.0) | 33 (30.8) | <0.001 | <0.001 |
| **Diagnostic group, n (%)** |  |  |  |  |  |
| Mild cognitive impairment | 142 (54.2) | 66 (43.4) | 76 (69.1) | <0.001 | <0.001 |
| Dementia | 120 (45.8) | 86 (56.6) | 34 (30.9) |  |  |
| **Cognition** |  |  |  |  |  |
| Education, years | 13 (11-16) | 13 (12-16) | 12 (10-15) | 0.043 | 0.065 |
| MoCA score | 20 (16-24) | 19 (16-24) | 21 (17-24) | 0.131 | 0.052 |
| MMSE score | 25 (21-28) | 24 (21-28) | 25 (22-29) | 0.484 | 0.333 |
| **CMBs, n (%)** |  |  |  |  |  |
| Present (at least one) | 83 (31.7) | 47 (30.9) | 36 (32.7) | 0.756 | 0.598 |
| One | 40 (15.3) | 20 (13.2) | 20 (18.2) | 0.562 | 0.747 |
| 2-4 | 16 (6.1) | 10 (6.6) | 6 (5.5) |  |  |
| 5-9 | 8 (3.1) | 6 (3.9) | 2 (1.8) |  |  |
| ≥10 | 19 (7.3) | 11 (7.2) | 8 (7.3) |  |  |
| Deep only | 8 (3.1) | 2 (1.3) | 6 (5.5) | 0.072 | . |
| **Comorbidities** |  |  |  |  |  |
| Hypertension, n (%) | 145 (57.8) | 88 (59.9) | 57 (54.8) | 0.424 | 0.492 |
| Stroke, n (%) | 57 (21.9) | 30 (20.0) | 27 (24.5) | 0.381 | 0.298 |
| HbA1c, % | 5.54 (5.31-5.81) | 5.45 (5.26-5.72) | 5.63 (5.35-5.99) | 0.074 | 0.164 |
| HbA1c ≥6.5%, n (%) | 24 (10.0) | 12 (8.4) | 12 (12.4) | 0.313 | 0.433 |

Data are presented as median (25th–75th percentile) or n (%). Unadjusted P values were calculated using Kruskal–Wallis tests for continuous variables, Pearson χ² tests for categorical variables, and Fisher exact tests for deep-only CMBs. Adjusted P values compare amyloid-positive and amyloid-negative participants after accounting for the following covariates: APOE4 carrier status: age; MCI versus dementia diagnosis: age and sex; education: age and sex; MoCA and MMSE: age, sex, and education; CMB presence and burden: age, sex, and hypertension; hypertension and HbA1c outcomes: age and sex; and stroke: age, sex, and hypertension. Age and female sex were not adjusted. Deep-only CMBs were not adjusted because of the small number of events. The CMB-burden P value shown is the global comparison across 1, 2–4, 5–9, and ≥10 CMBs among CMB-positive participants. Two-sided P < .05 indicated statistical significance.

**Missing data:** APOE4, 8; education, 47; MoCA, 43; MMSE, 92; stroke, 2; hypertension, 11; HbA1c, 22.

**Abbreviations:** Aβ, amyloid beta; MCI, mild cognitive impairment; MoCA, Montreal Cognitive Assessment; MMSE, Mini-Mental State Examination; APOE4, apolipoprotein E ε4 allele; CMB, cerebral microbleed; HbA1c, glycated hemoglobin.
