# Supplementary material for: Cerebral microbleed assessment on MRI in a memory clinic: Reliability, prevalence, and implications for anti‐amyloid therapy
Source: Alzheimers Dement (Amst). 2026 Sep 30;18(4):e70472. doi: 10.1002/dad2.70472 (PMC13627262; doi:10.1002/dad2.70472)
Supplement: Supplementary file 3 [file DAD2-18-e70472-s002.docx]

*Supplementary Figure 1. Susceptibility-weighted imaging (SWI) illustrating variability in cerebral microbleed burden. Left: Case with a single microbleed (white arrow). Right: Case with multiple microbleeds (10 selected lesions marked with white arrows).*
